# Supplementary material for: Mitochondrial dual-coding genes in Trypanosoma brucei
Source: PLoS Negl Trop Dis. 2017 Oct 9;11(10):e0005989. doi: 10.1371/journal.pntd.0005989 (PMC5650466; doi:10.1371/journal.pntd.0005989)
Supplement: S1 Fig — ATPase 6 (A), COIII (B), CR3 (C), CR4 (D), ND3 (E), ND7 (F), ND8 (G), ND9 (H), and RPS12 (I). Uppercase letters indicate nucleotides originally encoded in the DNA, lower case u's indicate uridines inserted during editing and asterisks indicate uridines removed during editing. (PDF) [file pntd.0005989.s001.pdf]

S1 Fig. Alignments of *T. brucei* and *T. vivax* edited mRNAs ATPase 6 (A), COIII (B), CR3 (C), CR4 (D), ND3 (E), ND7 (F), ND8 (G), ND9 (H), and RPS12 (I). Uppercase letters indicate nucleotides originally encoded in the DNA, lower case u's indicate uridines inserted during editing and asterisks indicate uridines removed during editing.

A. ATPase 6 - Pan-edited non-dual coding

```
AAAAAUAAGUAUUUUGAUAUUAUUAAAGUAAAuAuGuuuuuAuuuuuuuuuuGuGAuuuA T. brucei
---------------------------------AuGuuuuuGuuuuuuuuuuGuGAuuuG T. vivax

UUUUG-GuuGCGuuuGuuA----uuAuGuAuGuAuuAuuGuGuAuGAuCuAGGuuAuGuu T. brucei
UUUUG*GuuGCGuuuGuuA****UUAuGUGuGuAuuAuuGuGuGuGAuCuAGGuuAuGuu T. vivax

uuAuuGuGuAuuuuAA---uUGuuuAAuGuuGAuuuuuG-AuuuuuuAuuAuuuuGuuuG T. brucei
uuGuuGuGuAuuuuAA***UUGuuuGAuGuuAAuuuuuG*AuuuuuuGuuGuuuuGuuuG T. vivax

*UUUGAuuuGuAuuuGuuuGuuGGuuuGuG***UUUGuuuuuAuuGuuGuGGuuuAuGuu T. brucei
-uuuGAuuuGuAuuuGuuuAuuGGuuuAuG---uuuAuuuuuGuuAuuGuGGuuuAuGuu T. vivax

GuuuA----AuuuAuAuAGuuuAAUUUUGuAuuA*UUGuAuuAC----uUAUUUG***AA T. brucei
GuuuA****AuuuGuAuAGuuuGAUUUuGuAuuA*UUGuAuuACC****UAuuuG--*AA T. vivax

uuuG*UAuuUGuuGuuuuGuAuuGuuuuuuuuAuuGuA-----uAuuG-CAuuuuuAuuuu T. brucei
uuuG-uAUUUGuuGUUUuGuAuuGuuuuuuuuAuuGuA*****UA-uGuCAuuuuuGuuuu T. vivax

uGuuuuGuuuuuuA-uGuGAuuuuuuuuuuGuuuAAuAAuuuGuUA-GuuGGuGAuA**** T. brucei
uGuuuuGuuuuuuGuuG-GAuuuuuuuuuuGUUUAAuAGuuuGuuGUG-uGGuGAuA---- T. vivax

GuuuuAuGGAuG--uuuuuuuuAUUC**GuuuuuuGuuGuGuuuuuuAGAGuGuuuuuCu T. brucei
GuuuuAuGGAuGuuuuuuuuuuuG-*C--GuuuuuuGuuGuGuuuuuuAGAAuGuuuuuCu T. vivax

uuGuuGuGuC----GuuGuuuGuCGACGuuuuuGCGuuuG-UUUUGuAAuuuAuuAuCAu T. brucei
uuGuuAuGUC****GuuGuuuGuCAACAuuuuuACGuuuG*UUUUGuAAuuuAuuGuCAu T. vivax

CCCAuUUUUUAuuGuuGAuGuuuuuuG-A-uuuuuuuUAuuuuA-uuuuuGuuuuuuuuu T. brucei
CCCAUUUUuuAuuGuuAAuGuuuuuuGuAuuuuuuuuuUAuuuuAuuuuuuG-uuuuuuuu T. vivax

uuuA--------uG----GuGuuuuuuG--uuA-uuGAuuuAuuuuAuuuAuuuuuGuG- T. brucei
uuuGuuuuuuuuuGuuuuGuG-----uAuuuuAuuuGGuuuAuuuuGuuuG--uuuGUGu T. vivax

-uuuuGuuuuuGuuuAuuAuuuuAU--G-uGuuuuuAuAuUUGuuGGAuuuAUUuGCC-- T. brucei
uuuuuGuuuuuGuuuGuuGuuuuAUuuGuuG---uuGuAuuuAuuGGAuuuAuuuGCC** T. vivax

***GC-CA-uAuuAC****AGuuAuuuAuuuuuuGuAAuAuGAuuuuGCAGuuGAuAAuG T. brucei
***GC**GuuAuuAC----AGuuGuuuAuuuuuuGuAAuAuGAuuuuGCAAuuGGuAAuG T. vivax

--G**AuuuuuuGuuGuuuuuGuuG-uuuGuuuAGuuuuGuAuuuGAuuuuuGAuAGuuA T. brucei
**G**AuuuuuuAuuGuuuuuGuuGuuuuG-uuAG------------------------- T. vivax

uuAuAuuGuuGuuGAAAuuuG**GuuUGuuA**UUGGAGUUAUAGAAUAAGAUCAAAUAA T. brucei
------------------------------------------------------------ T. vivax

GUUAAUAAUA T. brucei
---------- T. vivax
```

B. COIII - Pan-edited non-dual coding

```
GGUUAUUGAGGAUUGUUUAAAAUUGAAUAAuuAuuAuuuuuuuAuGuuuuuGuuuC**** T. brucei
------------------------------------------------------------ T. vivax

*GuuGuAuAuuuGuuGGuGuuA-****GuGGuGuuuuuGuuuuuuuAuCuuuACCuGCCA T. brucei
------------------------------------------------------------ T. vivax

uuGuuAuuGuGuAuuGGuuAuuuuGuuuGuuG****GGAuuuAuuuGuuuAuuGUUUG** T. brucei
------------------------------------------------------------ T. vivax

**GuAGuuuuuuAuuuGuuGAuuGuG****GuuuuAuuuuuuuuuuuGuuGGuuuuuGuA T. brucei
------------------------------------------------------------ T. vivax

uuuGuuuGuuGuuGuuAuuGuuAGAuuuGuuuuGuGAuuuuuuACGuGGuuuAuuuGAuu T. brucei
------------------------------------------------------------ T. vivax

uuuGuGuuuuAuuACGuuGuAuCCAGuAuuGuuuuuuAuGGuuuuuAuGuAG*UGAGuuu T. brucei
------------------------------------------------------------ T. vivax

GuuuuAuuuAuGGCGuuuuuuG**UUGuAuuAuuuGGuuuAuGuuuAuuuuuGuGuuGuG T. brucei
------------------------------------------------------------ T. vivax

AGuuuGCUUUCGuuuuuuGuuuACCuuAuAuGuuuuGuuGuuuAuuAuGuGAuuAuGGuu T. brucei
------------------------------------------------------------ T. vivax

uuGuuuuuuAuuGG*UAuuuuuuAGAuuuAuuuAAuuuGuuGAuAAAuACAuuuuAUUUG T. brucei
------------------------------------------------------------ T. vivax

uuUGuuAGuGGuuuAuuuGuuAAuuuuuuuGuuuuGuGUUUUUGGuuuAGGuuuuuuuGu T. brucei
------------------------------------------------------------ T. vivax

uG**UUGuuGuuuuGuAuuAuGAuuGAGuuuGuuGuuuG****G--uuuuuuGuuuuuG- T. brucei
--------------------------------uuG-uuG--uuGuuuuuuuuGuuuuuGu T. vivax

uGAAACCA--GuuA---UGAGA**GUUUGCAuuGuuAuuuAuuACAuuAAGuuGuGG*** T. brucei
uGAA-uCA**GuuG***UGGGA**AuuuACGuuGuuAuuuAuuACGuuGAGuuGuGG--- T. vivax

*UG-uuuuuGGuuCuAuuuuAuuuuuAuuG---GAuuuAuUACAuuuuA**UGCAuGuuu T. brucei
-uG*UUUUUGGuuCuAuuuuAuuuuuAuuG***GAuuuGuuGCAuuuuA--uGCAuGuuu T. vivax

uuuuAGGuGuuuuGuuGuuG-uuuAuuuG-uuuuAuG--CGuuuGuuuAAuuuuuuGuGu T. brucei
uuuuAGGuGuuuuAuuGuuGuuuuA--uGuuuUUAuG**CGuuuGuuuAGuuuuuuAuGu T. vivax

AuGGAuACACGuuuuGuuuuuuuGuAuuGuGuuuGuuuAuAuuGACAuuuuGuuGA-UUU T. brucei
AuGGAuACACGuuuuGuuuuuuuGuAuGuuGuuuGuuuGuAuuGACAuuuuGuuGAUUU* T. vivax

AGuuuGAuuuuuuuuAuuGCGAuuuGuuuAuuuuGAuGuuuuAuG----uGuuAuGuAuu T. brucei
GGuuuGGuuuuuuuuGuuGCGAuuuGuuuAuuuuGAuGuuuuAuG****UGuuAuGuAuu T. vivax

uGuGuGuGuAAuuuuAuuGGuGuuuuUUUAGUUGuuGAuuA*GuuAAuuuGuAuuGGUAG T. brucei
uGuGuGuGuAG------------------------------------------------- T. vivax

UUUGUAGGAAG-- T. brucei
------------- T. vivax
```

C. CR3 - Pan-edited dual coding

```
AGAAAUAUAAAUAUGUGUAUGAUAUAUAAAAACAAuGuuuGA****UUGuuuGGuuuuG- T. brucei
----------------------------------AuGuuuGA---*UUGuuuAGuuuuGU T. vivax

--uuG-uuuuUUUAuuGuuuGuuuGuACAuuuuuuuuGuuuuuuAuuuGuuuGuG----- T. brucei
U***GuuuuuuuuAuuG-uuGuuuGuACAuuuuuuuuGuUUUUUGuuuAuuuGuG***** T. vivax

***A**UUUGuuuuuAuGuuuGuuA-*UUUA------GuuuuuGuuuuuuAuuGGAuuuu T. brucei
***A--uuuGuuuuuAuGuuuGuuG--uuuGuuuuuuG--uuUA---uuuG-uGGAuuuu T. vivax

uGuuuuuuAuuuAA----uA--uGGGuuuA---uUGuuGuGuuuAuuuuuuuuuuuuAuu T. brucei
uGuuuuuuGuuuAA****UA**UGGGuuuG***UUGuuGUGuuuAuuuuuuuuuuuuGuu T. vivax

uuAuCAuuuGAuAuGuuGuuAuCAuuuuUAuuAuuGuAuAuAAGuUUUCGUUAUUAGAUU T. brucei
uuAuCAuuuGAuAuGuuAuuAuCGuUUUUGuuAuuAuAuAuAAGuUUUCGUUAUUAA--- T. vivax

AAAAAAGUAUGCAAAUAAUUUUUGU T. brucei
------------------------- T. vivax
```

D. CR4 - Pan-edited dual coding

```
UAAUUUAUUGUUAUCUUUGUGUAUUUAUUAuuAuuuuAuuuuAA---uuuuG---GuuGu T. brucei
-------------------------------------------AUUuuuuuGuuuGuuGu T. vivax

GC--***AuuuuuuuuuuuuuuAuuuG***GuG*UGuuuGuGuuuuA*UGuA*C*A---- T. brucei
GC*****AuuuuuuuuuuuuuuAuuuG---GuG-uGuuuGuGuuuuA-uGuA--uA**** T. vivax

*GuuuAuGGuAuAuuuuAuuGuuGuuuuGuuuuuuGuuuuuGuuGUUUG-uuUGuG--uG T. brucei
*GuuuGuGGuAuAuuuuGUuGuuGuuuuuuuuuuuGuuuuuuuuGuuuG***UGuGuuuG T. vivax

GGuA--uGuuuuAuuuGuuuuGuuAuA----GuuGuuuGuuuuuuuuuGuuGuUUUG*GG T. brucei
GGuA**UGuuuuAuuuGuuuuGuuAuA****GuuGuuuGuuuuuuuuuuuuGuuuuG-GG T. vivax

uuGuG----AuuuuuuAuuG**G--uGuuuuG***AuuGuAuA---GuuuAuuuuuuuuG T. brucei
uuGuG----AUUUUuuGuuA--GuuuA--UuG---AuuGuAuA***GUuuGuuuuuuuuG T. vivax

uGAC-GuuAuAAuuUUGuuuAuuuuuuuuuuuuAuuuuGuuuuGuGuuuuuuG---uAuu T. brucei
uGAC*GuuAuAAUuuuGuuuAUUUUuuuuuuuuGUuuuGUUuuGuGuuuuuuGuuuuGuu T. vivax

G*UUGuuuuuA-uUUGGuuuGuuuGGuuuuuuuuuG***UAuuuuuuGUUGuGuuuuGuG T. brucei
GuuuuuuuuuG----GUUU*GuuuGGuuuuuuuuuG---uAuuuuuuGuuAuGUuuuGuG T. vivax

uuAuuuuuuGAuuuAuuuuuuAuGuUGuuuuuUGuuuuG--GG***UG*G-uuuuuuuGu T. brucei
uuAuuuuuuGAuuuGuuuuuuAuGuuGuuuuuuG---uAuuGG----GUGGuuuuuuuGu T. vivax

uuuuGuuuuuuuuuuuuGuuuAuGuuuGuuuuuA---uuuGuGGuuGuuG--uuAuuuuG T. brucei
uuuuGuuuuuuuuuuuuGuuuAUGuuuA---uuGuuuuuuGuAGuuAUUG**UUGuuuuA T. vivax

uuAGuuuGGuuGuuGUUG-uuAuuUGuG---uA--uA****GGUUUAuuUAuA*UGCGuu T. brucei
uuAGuuuGGuuGuuGuuGUU*AuuuGuGU***AUuuA--UUGGuuuAuuuAuA-uGCG-- T. vivax

uuuuAuuuuA------GAuAAuUAuG****G****UA**UUGGUUUUAUAAAAUGUUUUU T. brucei
uuuuG---uGUUUU**AA------------------------------------------ T. vivax

UCU T. brucei
--- T. vivax
```

E. ND3 - Pan-edited dual coding

```
UCAAAAAAUCCUCGCCUUUUUACUUUA-GUUUGUUAUCAuuA--uuuuuAuAuuuGuuuu T. brucei
-------------------------AUG----UUAuCA--AUuuuuuuGuAuuuG---- T. vivax

UG---*A*UA-uuGuGGuuuA**UUAuuuuAuuuA-uAGG--uuuuuuuuuAuGuuuuuu T. brucei
-GUUUuG-uGuuuGuGGuuuA--UuAuuuuA-uuGuuAGGuuuuuuuCU**AuGuuuuuu T. vivax

AuGuuuuuuAuuGCAuuuuuuuG----AuuGuuuuCGuuGuuGuuuGuGGuuuuCGuGuG T. brucei
AuGuuuuuuGuuACAuuuuuuuG****AuuGuuuuCGuuGuuGuuuAuGAuuuuCAuGuG T. vivax

---GuUUGuA---uGAuAuG--A-----AuUCACGuuuG*GUGuuuuA--uACAuuGGAu T. brucei
***GuuuGuA***UGAuAuG**A*****AuuCACGuuuG-GuGuuuuA**UACAuuAGAu T. vivax

uuAUGuuuuGuuA------------GuUGuUUGuuuuuuGuAuuGuu---AAAuuCCAu T. brucei
uuAuGuuuuGuuA************GuuGuuuGUuuuuuGuGuuAUU***GAAUuCuGu T. vivax

UAuuuGu----GuUUUGuuGuuuGuuuuuG--UG----------A-uA*GuGuuGuuuuA T. brucei
uAuuuGU****GuuuuGuuAUUUG----uAuuuGU********GuuG-GuAuuGuuuuA T. vivax

uuuuuGuuAU----GGuuuuuuG-uUUUGuGGuuuuuGuuuuuuGuuG-uA-uGuA--- T. brucei
uuuuuGUUAU****GGuuuuuuA*UUUUGuGGuuuuuGuuuuuuG-uGuuGuuA--**U T. vivax

uAG****GAuuUGuGuGGuAuuuuuGGGA-UCAC*GuAuAuuuGuGuGGuGUAAuuuuAu T. brucei
UAG----GGuuuGuGUGGuAuuuuuGAGA*UCA-uGuAUAUU----------------- T. vivax

uuuGuuuAuGA**UGuuuUUUGUUGUAUUAUACAUAUUAUAUUAAUAAAUAUAUAAAA T. brucei
---------------------------------------------------------- T. vivax
```

F. ND7 - Pan-edited dual coding

```
UGAUACAAAAAAACAUGACUACAUGAUAAGUAuCAuuuuAuG-uuAuuuuuG--GuAGuu T. brucei
--------------------------------------AuGuuuAuuuuuGuuGuAGuu T. vivax

uuuuuACAuuuGuAuCGuuuuACAuuuG*GUCCACAGCAuCCCG***CAGCACAuG**G- T. brucei
uuuuuGCAuuuGuAUCGUuuuACAUuuG-GCCCACAGCAuCCCG---CAGCACAuG-*G* T. vivax

uGuuuuAuGuuGuuuAuuGuAuuuuuGuGGuGA*AuuuAuuGuuuA**UA---UUGAuUG T. brucei
UGuuuuAuGUuGuuuAUUGuAuuuuuGUGGuGA-AuuuAuuGuuuA--uA***UUGAuuG T. vivax

uAuuAuA***G*GuuA--UUUGCAUCGUGGUACAGAAAAGUUAUGUGAAUAUAAAAGUGU T. brucei
uAuuAuA---G-GuuA**UUUGCAUCGAGGUACAGAAAAGUUAUGUGAGUAUAAGAGCGU T. vivax

AGAACAAUGUCUUCCGuAUUUCGA---CAGGUUAGAuuAuGuuA---*GuGuuuGuuGuA T. brucei
AGAGCAGUGUCUUCCGuAUUUuGAU***AGAuuAGAuuAuGuuA****GuGuuuGuuGuA T. vivax

AuGAGCAuuuGuuGuCuuuA***UGuuuuGAGuA--uAuGuuGCGAuGuuGuuuGuCGuu T. brucei
AuGAACAuuuAuuGuCuuuA---uGuuuuGAGuA**UAuGuuACGGuGuuGuuuAuCAuu T. vivax

ACGuuGuGCAuuuAuGCGuuuAuuAAuuGuA****GAAuuuAC***CCGuAGuuuuAAuG T. brucei
GCGuGuuGCAuuuAuGCGuuuAuuGAuuGuA----GAGuuuACU**C*GUAGuuuuAAuG T. vivax

GuuuGuuGuGuAuAuCAuGuAuGGuuuuGG*AuuuAGGuuGuuuGuCUCCGuuG*UUAuG T. brucei
GuuuAuuGuGuGuGuCGuGuAuGAuuuuAG-AuuuAGGuuGuuuAuCCCCGuuA-UuAuG T. vivax

AuCAuuuGAGGAA-***CG*UGA-CAAAuuGAuGACAuuuuuuGAuuuAuG**UUGuGGu T. brucei
GuCAuuuGAGGAG****CG-uGAU*AAGuuAAuGACGuuuuuuGAuuuGuG--uuGuGGu T. vivax

uGuCGuAuGCAuuuGGCUUUCAuGGuuuuAuuA-*GGuAUUCUUGAUGAuuuuGuuuuuG T. brucei
uGuCGuAuGCAuuuGGCUUUCAuGGuuuuAuuG**GGuAuuCUUGAuGAuuuuGuuuuuG T. vivax

GuuuuGuuGAuuuuuuGuuGuuGuuGA***UAAuAuCAuGuuuGuuuGuuAuGGAuuGuu T. brucei
```

GuuuuGuuGAUUUuuuGuuGuuAuuGA---uAAuAuCGuGuuuGuuuGuuAuGGAuuGuu T. vivax

AuGAuuuGuuAuuuG--uGGGuAA---UCGuuuAuuuUAuuuGCGuuuGC***GuGGuuu T. brucei
AuGAuuuAuuGuuuG**UGGGuAA***UCGuuuGuuuuAuuuGCGuuuGC---GuGGuuu T. vivax

GuCAuuuuuuuGAuuuA---uAuGAuuuA**GuuuuuA**A**UAGuuuAAGuGGuGuuuu T. brucei
GuCAuuuuuuuGAuuuG***UAuGAuuuG--GuuuuuA--A--uAGuuuGAGuGGuGuuuu T. vivax

GuCuCGuuCGuuAGGuAuGGuGuGAGAuuGUCGuuuAuuuAGuuGuuA****UGA***** T. brucei
GuCACGuuCAuuGGGuAuGGuAuGAGAuuGCCGuuuAuAuuGuuGuuA-***UGA----- T. vivax

GuUG-uAuuuuA---uGuuuuGuuAuGAuuAuuGuuuuuGuuuuAuA-GGuGAuGCAuuu T. brucei
GuuG*UAUUUUA***UGuuuuGuuAuGAuuAuuGuuuuuGuuuuAuA*GGuGAuGCAuuu T. vivax

GA*UCGuuuAuuuuuACGuuuGuuuGAUAuGCGuAuGAGuuuGuuGAuuuGuAAGCAA-u T. brucei
GAC*CGuuuGuuuuuGCGuuuGuuuGAuAuGCGuAuGAGuuuGuuGAuuuGuAAGCAA*U T. vivax

GuuuuuuuGuuGGuuuuuuuGuuuuuG*****GuuuuGuuuGuuuGuuuG**AuuAuuuA T. brucei
GuuuuuuuGuuGGuuuuuuuGuuuuuG-----GAuuuGuuuGuuuGuuuG--AuuAuuuG T. vivax

uAuuGuGAuAuuACCAuuG****AGACCAuuAuuAuGuuAuuuuAuAGuuuG--uGGuGu T. brucei
uAuuGuGAuGuuACCAuuG----AGACuAuuAuuAuGuuGuuuuAuAGuuuA**UGGuGu T. vivax

uGuuGuuuGCCGGGuAuAU*-----CAuuuGC*UUGU-GuuGAACACCCCAAAG-----G T. brucei
uGuuGuuuACCAGGuAuAU******CAUUUGC-UUGU*GuuGAGCAuCCCAAGG*****G T. vivax

uGA***GuAuuGuuuGuuAuuAU****GuuuuuGuGuuGGuuuAuGuuCUCGuuuACGuu T. brucei
uGA---GuAuuGuuuGuuAuuAU****GuuuuuGuGuuGGuuuGuGUUCCCGuuuGCGuu T. vivax

uGCGuuGuGCGGAuuuuuuGCA--*UA--UUUGuuuAuuGGAuGuuuGuuuGCGuGGuuu T. brucei
uGCGuuGuGCGGAuuuuuuACA***UA**UUUGuuuGuuGGAuGuuuGuuuACGuGGuuu T. vivax

uuuAuuGCAuGAuuuAGuuGC--***C*GuuuuA--GGuAAuAuuGAuGuuGuuuuuGGA T. brucei
uuuAuuGCAuGAuuuAGuuGC*****C*G--uuAuuGGuAAuAuuGAuGuuGuuuuuGGA T. vivax

uCC--GUAGAUCGuuA*GuuuuAuAuGuG**A******GGUUAUUGuAGGAUUGUUUAAA T. brucei
uCU**GuGGAUCGuuA*G-- T. vivax

AUUGAAUAAAAA T. brucei
------------ T. vivax

G. ND8 - Pan-edited non-dual coding
-------CAAUUUAAUAAUUUUAAGUUUUGGUUGAUUAuuAuuuuuuuAuuuuuuuAuuu T. brucei
-- T. vivax

uuGuAuGuuuuuuuuuGAuuuuuuGuuuuuuuUUUUUGuuuGuuuuuAuAuGuGUuuuGu T. brucei
----AuGuuuuuuuuuGAuuuuuuGuuuuuuuuuuuuGuuuGuuuuuAuAuGuGuuuuGu T. vivax

uuGuuGuGuuA****CuA*UUUGuuuA-***CCCAuuGAGuuAACCA--uuGuuAGuuuA T. brucei
uuGuuGuGuuA---CC-A-UUuGuuuA****CCCAuuGAAuuAAC-AuuuuG-uAGuuuG T. vivax

uuGGuuC--GuGGUAA---C-C---AuuuuuuGCGUUUUuA***UUGGuGuGGuuuAGAG T. brucei
uuGA-*CCCGuGGuAA***C*C***AuuuuuuGCGuuuuuA--*UUGAuGuGGuuuAGAA T. vivax

CGuuGuAuuGCuuGuCGuuuAuGuGAuuuAAuuuG-C-----CCuA****GuuuAGCAuu T. brucei
CGUuGuAuuGCuuGuCGuuuAuGuGAuuuGAuuuGuC*****CC-A----GuuuAGCAuu T. vivax

GGAuG***UUCGuGuuGGGuGG---AGuuuuGGuGGuCA**UC*GuuuuGCG--GAuuG- T. brucei
AGAuG---uuCGuGuuGGGuGG***AGuuuuGGuGGuCA--uC-GuuuuGCA**GAuuG* T. vivax

```
-AuuuACAuuGAGuuA-*UC**GU-**C----GuuGuAuuuAuuGuGGuuuuuGuAuGCA T. brucei
*AuuuACAuuGAGuuA***C-CG***AC****GuuGuAuuuAuuGuGGuuuuuGuAuGCA T. vivax

uGuuuGCCCGACAGAU****G---CC----AuuA-----CGCA---UUCAuuGuuuGuuA T. brucei
uGuuuGuCCAACAGAu----G***CC****AuuA*****CACA***UUCAuuGuuuGuuA T. vivax

uGuGuuuuuGuuGuuuA-------GCC**A**UGuAuuuAuuG*GCGC***C***C---- T. brucei
uGuGuuuuuGuuGuuuA*******GCC**A--UGuAuuuAuuG-GCGC---C---C**** T. vivax

AAGuuuuuAuuGuuuGG---uuGuuGuuuuAuGuuAuuuGAuuuuuAuuuGuGuuuuGuG T. brucei
AAGuuuuuGuuAuuuGG***UUGuuGuuuuAuGuuGuuuGAuuuuuAuuuGuGuuuuGuG T. vivax

uAGuuAuuuAuuuuGGGuGAuuuAuuGUGuuuAuGAuuuAA***AGAA**AuuCACGGUG T. brucei
uAG--------------------------------------------------------- T. vivax

AAAUUAAAUUUUGACUAAAU T. brucei
-------------------- T. vivax
```

H. ND9 - Pan-edited dual coding

```
UUAAUAUCAACUUAAUUUUUUUUAUAAACAuuAuAuuAUGuGuA--uAuUUUUAuGuuuA T. brucei
-------------------------------------AuG-G-GuuuGuuGuuGuGuuuA T. vivax

uuuCGuuuAuGuuuuuGuuuAAuuUUAuuuuA**UUGuuuGuGuuGuA----------GA T. brucei
uuuCGUUuGuGUUUUUGuuuGAUuuuGuuuuA--UuGuuuGUGuuGuA**********GG T. vivax

uGGuGuuUUGuuuGuuuuGuuGA-uuGuAGuuuuuuGuuuuuuuAuuGuuuuGuuA-Guu T. brucei
UGGuGuuuuGuuuGuuuuGuuGA*UUGuAGuuuuuuGuuuuuuuAUUGUUUuGuuA*Guu T. vivax

uuuuuuuGuuuuAUUGuAuGuuuuuAuuuuuuAAuuuGuG-AuuuuuGuuuuuAuAuuGU T. brucei
uuuuuuuGuuuuAuuGuAuGuuuuuAUUuuuuAAuuuAuG*GuuuuuGuuuuuGuAuuGu T. vivax

UGuG-AuUUGuuAuuGAuuGAuuuuuGuGGuuuuuGuuuuuGuCGuuuuAuGuuGuuGUA T. brucei
uGuG*AuuuAuuGuuGAuuGAuuuuuGuGGUuuuuGuuuuuGuCGuuuuAuGuuAuuAuA T. vivax

uAuuuuAuuuuGuuuGuuuuuGuGuGuuCGuuuGuGuuuuGuuuuGuGuuGUUUGuuuGU T. brucei
uAuuuuGuuuuGuuuGUuuuuGuGuuuuCGuuuAuGuuuuGuuuuGuGUUGUUuGuuuuu T. vivax

AuuuuuuGGAuuG----uGuuuuA-*GuuuuA**GuuGuuuuuGuUAuGC---GuuuuuG T. brucei
GUUUUuuGGAuuG****UGuuuuA**GuuuuA--GuuGuuuuuGuuAuGC***GuuuuuA T. vivax

uuGuuGGA----ACGC*GAAuGuuuUGAUUUGuuuGGuuuuUAuuuuG--uuGGuAAuGA T. brucei
UUGuuAGA****ACG-uGAGuGuuuuGAuuuGuuuGGuuuuuAuuuuG**UUGGuAAuGA T. vivax

uAuuuuACAUCGuuuAuuuGuuG--AuuG****GuuuuuuGuuG-GuuuuuuuuuGuuGA T. brucei
uGuuuuACACCGuuuAUuuGuuG**AuuG----AUUuuuuGuuG*GuuuuuuuuuGuuGA T. vivax

-AGuGuuAUCCA--uuAuuuGGuuuGuuuGuAuuGuuAuuuuGuG---uGuuG**GuG-G T. brucei
*AGuGuuAuCCA**UUAuuuGGUuuGuuuGuAuuAuuGuuuuGuGuuuuA--GuuA-AuG T. vivax

A--GGA-GAUA-GuAuGuACGuuuACAAuGuuA--uuuuuGuuGuuGCAuACC**AAuuU T. brucei
A-**GAUGAuAuGuA----CGuuuACAAuG--GuuuuuuuGuuGuuGCAuACC**AAuuu T. vivax

UUAuuuG*CA-----uuAuuuuAuuuA***AuA**UCACCGuUGUAAUUCUAAAUUUCUC T. brucei
uuAuuuG-CAUUuuuuuG-uuuA--*G--------------------------------- T. vivax

ACUUCC T. brucei
------ T. vivax
```

I. RPS12 - Pan-edited dual coding

```
CUAAUACACUUUUGAUAACAAACUAAAGUAAAuAuAuuuuGuuuuuuuuGCGuAuGuGA* T. brucei
-----------------------------------------------------AuGuGA* T. vivax

UUUUUGUAUG*GuuGuuGuuuAC----*GuuuuGuuuuAuuuGuuuuAuGuuAuuAuAuG T. brucei
UUUUUGuAuG-GuuGuuGUUUGC*****GuuuuGuuuuGuuuGuuuuAuGuuAuuAuAuG T. vivax

AGuCC----G**CGAuuGCCCAGuuCCGGuAACCGACGuGuAuuGuAuGC**C****GuA T. brucei
AGUCC****C--CGAuuGCCCAGuuCCGGuAAuCGACGuGuGuuGuAuGC--C--**GuG T. vivax

uuuuAuuUAuAuAAuuuuGuuuG-GA-uGuuGCGuuGuuuuuuuuGuuGuuuuAuuG--- T. brucei
uuuuAuuuGuAuAAuuuuG--uGuGGuuGuuGCGuuGuuuuuuuuGuuG---uG-uGUUU T. vivax

---GuuuA---GuuA--uG**UCAuuAuuuAuuAuAGA--***G----GGUGGuGGuuuu T. brucei
UuuG---GuuuG-CAUUUG--UCGuuAuuuAuuAuAGA*****G****GGuGGuGGuuuu T. vivax

GuuGAuuuACCC--***G****GuG*UAAAGuAuuAuACA*CG**UAuuG--uA--AGuu T. brucei
GuuGAuuuACCC*****G--**GuA-UAAAGuAuuAuACA-CG--uA-uGuuuAuuAAuu T. vivax

AGA*UUUAGAuAUAAGAUAUGUUUUU T. brucei
AA------------------------ T. vivax
```
