# Supplementary material for: Mitochondrial dual-coding genes in Trypanosoma brucei
Source: PLoS Negl Trop Dis. 2017 Oct 9;11(10):e0005989. doi: 10.1371/journal.pntd.0005989 (PMC5650466; doi:10.1371/journal.pntd.0005989)
Supplement: S2 Fig — A: CR3, B: CR4, C: ND3, D: ND7 5' Editing Domain E: ND9, F: RPS12, G: ND8 (Nondual-coding). Absent sequences were unavailable. All ORF alignments show published protein sequences. All ARF alignments show +1 or +2 (ND7 only) reading frame translations of the full length mRNA sequences. In the ARF alignments of CR3, ND7 and RPS12, translations were made using the alternative T. brucei mRNA sequences shown in Fig 1. Two alignments of CR3 ARFs are presented to display the P. serpens +2 reading frame which has no stop codons. L. tarentolae CR4 published protein sequence shows limited homology in the C terminus to all other CR4 protein sequences. The edited mRNA has two editing sites where 13 U residues are inserted. If the second of these insertion sites is shortened to 12 U residues, the translation of this mRNA has much better homology to other CR4 proteins. Alignments with translations of the two different sequences (13U and 12U) are both shown, with the location of the altered site highlighted in red. While ND8 does not appear to be dual-coding, this alignment was included as well, for comparison of the conservation of a nondual-coding gene with that of the dual-coding genes. It should be noted that ND8 is the only nondual-coding gene that is pan-edited in L. tarentolae, L. amazonensis, and P. serpens; ND7, A6 and COIII are only partially edited in these species. The internal M observed in the ND8 sequence is highlighted in yellow (see text).! = Termination codon. (PDF) [file pntd.0005989.s002.pdf]

S2 Fig. Alignments of protein sequences of pan-edited dual-coding genes in *L. tarentolae*, *L. amazonensis*, *P. serpens*, and *Perkinsela CCAP1560/4* with *T. brucei* and *T. vivax* sequences. A: *CR3*, B: *CR4*, C: *ND3*, D: *ND7* 5' Editing Domain E: *ND9*, F: *RPS12*, G: *ND8* (Nondual-coding). Absent sequences were unavailable. All ORF alignments show published protein sequences. All ARF alignments show +1 or +2 (*ND7* only) reading frame translations of the full length mRNA sequences. In the ARF alignments of *CR3*, *ND7* and *RPS12*, translations were made using the alternative *T. brucei* mRNA sequences shown in Fig 1. Two alignments of CR3 ARFs are presented to display the *P. serpens* +2 reading frame which has no stop codons. *L. tarentolae* CR4 published protein sequence shows limited homology in the C terminus to all other CR4 protein sequences. The edited mRNA has two editing sites where 13 U residues are inserted. If the second of these insertion sites is shortened to 12 U residues, the translation of this mRNA has much better homology to other CR4 proteins. Alignments with translations of the two different sequences (13U and 12U) are both shown, with the location of the altered site highlighted in red. While *ND8* does not appear to be dual-coding, this alignment was included as well, for comparison of the conservation of a nondual-coding gene with that of the dual-coding genes. It should be noted that *ND8* is the only nondual-coding gene that is pan-edited in *L. tarentolae*, *L. amazonensis*, and *P. serpens*, and *ND7*, *A6* and *COIII* are only partially edited in these species. !=Termination codon

A. CR3

CR3 ORF Alignment

```
CR3ORF T. brucei        --MFDCLVLL-FFYCLFVHFFCFLFVCDLFLCLLFSFCFLLDFCFLFNMGLLLCLFFFFI
CR3ORF T. vivax         --MFDCLVLL-FFLLLFVHFFCFLFICDLFLCLLFVFCLFVDFCFLFNMGLLLCLFFFFV
CR3ORF L. amazonensis   --MFDFVIIMFL-FMSFVHFFCFLFIVDLLFCLMFFVFFLYDFCFVCNLGFCCCLFFFFL
CR3ORF P. serpens       IFLFDFVLFLVLFLLFFVHFFCFLFIIDLFCCFLLLFFLVFDFCFCCCFGFVSCLFLFFV
                          :** :::: :    *********: **: *::: . :. ****   :*:  ***:**:

CR3ORF T. brucei        LSFDMLLSFLLLYISFRY!
CR3ORF T. vivax         LSFDMLLSFLLLYISFRY!
CR3ORF L. amazonensis   LSIDMILSFILLYVSFRY!
CR3ORF P. serpens       FHFDMVLSFILLFVSFRY!
                        : :**:***:**::****
```

CR3 ARF Alignment with P. serpens +1 RF

```
CR3ARF T. brucei         ---------RNINMCMIYKNNVYVVVLFWFWLYIFFVFYLFVICFYVCYLVFVFYWIFVF
CR3ARF T. vivax          --------------CLI----V!FCCFFYCCLYIFFVFCLFVICFYVCCLFFVYLWIFVF
CR3ARF P. serpens +1     !!VYNI!KHNILYFCLI---LFCF!CYFYCFLCIFFVFYLLLICFVVFYYCFF!CLIFVF
CR3ARF L. amazonensis    --K!NNMY!V!IYICLI----SLL!CFCLWVLYIFFVFYLLLICYFVWCFLFFFYMIFVL
                                       *:*      .       * ***** *::**: *    *.   ***:

CR3ARF T. brucei         YLIWVYCCVYFFFLFYHLICCYHFYYCI!VFVIRLKKYANNFC-
CR3ARF T. vivax          CLIWVCCCVYFFFLFYHLICYYRFCYYI!VFVI-----------
CR3ARF P. serpens +1     VVVLVLWVVYFYFLFFILIWFYLSYYYL!VSVIKSI!KHKLIS-
CR3ARF L. amazonensis    CVI!VFVVVCFFFFCYPLIWFCRLFYYMLVSDIKII!LLFL!!K
                          :: *   * *:*: : **      * : *  *
```

CR3 ARF Alignment with P. serpens +2 RF

```
CR3ARF T. brucei         -------RNINMCMIYKNNVYVVVLFWFWLYIFFVFYLFVICFYVCYLVFVFYWIFVFYL
CR3ARF T. vivax          ------------CLI----V!FCCFFYCCLYIFFVFCLFVICFYVCCLFFVYLWIFVFCL
CR3ARF P. serpens +2     ----------SKCIIYKNIIFYI-FVWFC----FVFSVIFIVFCAFFLFFIYYWFVLLFF
CR3ARF L. amazonensis    K!NNMY!V!IYICLI----SLL!CFCLWVLYIFFVFYLLLICYFVWCFLFFFYMIFVLCV
                                     *:*         :        *** ::.* : .  :.*.:  :.:: .

CR3ARF T. brucei         IWVYCCVYFFFLFYHLICCYHFYYCI!--------VFVIRLKKYA--NNFC------
CR3ARF T. vivax          IWVCCCVYFFFLFYHLICYYRFCYYI!--------VFVI------------------
CR3ARF P. serpens +2     IIVFFSVWFL--FLLLFWFCELFIFIFCFSFWYGFIFHIIICKFPLLKAFKNISL!V
CR3ARF L. amazonensis    I!VFVVVCFFFFCYPLIWFCRLFYYML--------VSDIKII!LL--FL!!K-----
                         * *   * *:     *:   .:   :         :  *
```

B. CR4

CR4 ORF Alignment with L. tarentolae 13U translation (Published Sequence)

```
CR4ORF T. brucei          ILILVVHFFFFYLVCLC-----FMYSLWYILLLFCFLFLLFVCVGMFYLFCYSCLFFFVV
CR4ORF T. vivax           IFLFVVHFFFFYLVCLC-----FMYSLWYILLLFFFLFFLFVCLGMFYLFCYSCLFFFFV
CR4ORF L. tarentolae 13U  -------------KCCCFWFFYVLFCVLYILFLFFFLFIWFVCYGLFYLYCICLFICFSL
CR4ORF L. amazonensis     ---ISNILLFLYIFIYICWLIF-MYSCWYILILFFFLFLLFVVYGLFYLYCIVCLFILCL
                                                 ::.  ***:** ***: **  *:***:*   :: : :

CR4ORF T. brucei          LGCDFLLVFWLYSLFFLWRYNFVYFFFLFCFVFFVLLF-LFGLFGFFLYFLLCFVLFFDL
CR4ORF T. vivax           LGCDFLLVYWLYSLFFLWRYNFVYFFFLFCFVFFVLLF-FFGLFGFFLYFLLCFVLFFDL
CR4ORF L. tarentolae 13U  LCCDFVVVFWLYSVFFVYRYNYFFFFVYFLGVYFFVIILICIWFFIFFFLCLCFDFL--F
CR4ORF L. amazonensis     LCCDFVVVFWLYSVFFIYRYNFVFFFFFLWFVFIFLIIFIFGFGFLFFFLVLCLVFYFEF
                          * ***::*:****:**::***:.:**. :  *::.::: :     :*::: **: :   :

CR4ORF T. brucei          FFMLFFVLGGFFVFVFF---------FCLCLFLFVVVVILLVWLL-----LLFVYRFIYM
CR4ORF T. vivax           FFMLFFVLGGFFVFVFF---------FCLCLLFFVVIVVLLVWLL-----LLFVYRFIYM
CR4ORF L. tarentolae 13U  WIFVYVVFCFLWIFVVCDVYFIFYIIFCFNCVGVLLVVIYICVSIFLYDVLYFNFNWIIL
CR4ORF L. amazonensis     LFMLFFVFCGFLLFVMFILFFVSFF---------VLIVLLFCWMLF-----IFVFRFICM
                           ::::.*:  : :**.                  :::*: :   :       * :.:* :

CR4ORF T. brucei          RFLF!
CR4ORF T. vivax           RFVF!
CR4ORF L. tarentolae 13U  KF---
CR4ORF L. amazonensis     RFVF!
                          :*
```

CR4 ORF Alignment with L. tarentolae 12U translation (Hypothetical Sequence)

```
CR4ORF T. brucei          ILILVVHFFFFYLVCLC-----FMYSLWYILLLFCFLFLLFVCVGMFYLFCYSCLFFFVV
CR4ORF T. vivax           IFLFVVHFFFFYLVCLC-----FMYSLWYILLLFFFLFFLFVCLGMFYLFCYSCLFFFFV
CR4ORF L. tarentolae 12U  -------------KCCCFWFFYVLFCVLYILFLFFFLFIWFVCYGLFYLYCICLFICFSL
CR4ORF L. amazonensis     ---ISNILLFLYIFIYICWLIF-MYSCWYILILFFFLFLLFVVYGLFYLYCIVCLFILCL
                                                 ::.  ***:** ***: **  *:***:*   :: : :

CR4ORF T. brucei          LGCDFLLVFWLYSLFFLWRYNFVYFFFLFCFVFFVLLFL-FGLFGFFLYFLLCFVLFFDL
CR4ORF T. vivax           LGCDFLLVYWLYSLFFLWRYNFVYFFFLFCFVFFVLLFF-FGLFGFFLYFLLCFVLFFDL
CR4ORF L. tarentolae 12U  LCCDFVVVFWLYSVFFVYRYNYFFFFVYFLGVYFFVIILICIWFFIFFFYVCVLIFYFEF
CR4ORF L. amazonensis     LCCDFVVVFWLYSVFFIYRYNFVFFFFFLWFVFIFLIIFIFGFGFLFFFLVLCLVFYFEF
                          * ***::*:****:**::***:.:**. :  *::.::::      :*:: :  ::::*::

CR4ORF T. brucei          FFMLFFVLGGFFVFVFFFCLCLFLFVVVVILLVWLLLLFVYRFIYMRFLF!---------
CR4ORF T. vivax           FFMLFFVLGGFFVFVFFFCLCLLFFVVIVVLLVWLLLLFVYRFIYMRFVF!---------
CR4ORF L. tarentolae 12U  LFMLFFVFCGFLLFVMFILFFISFFVLIVLVFCWLLFIFVFRFFCMTFCILILIGLF!NL
CR4ORF L. amazonensis     LFMLFFVFCGFLLFVMFILFFVSFFVLIVLLFCWMLFIFVFRFICMRFVF!---------
                          :******: **::**:*: : : :**::*::: *:*::**:**: * * :
```

CR4 ARF Alignment with L. tarentolae 13U translation (Published Sequence)

```
CR4ARF T. brucei           -----IYCYLCVFIIILF!FWLCIFFFFIWCVCVLCTVYGIFYCCFVFCFCCLFVWVCFI
CR4ARF T. vivax            -----------------FFCLLCIFFFFIWCVCVLCIVCGIFCCCFFFCFFCLCVWVCFI
CR4ARF L. tarentolae 13U   QIH!NTYMYNCKSVV-VFGFFMY---------YFVC---CIFYFCFFFCLFDLCVMVYFI
CR4ARF L. amazonensis      ------DIKNIK!VI-FYYFYIFL---FTFVGWFLCIVVGIFWFYFFFYFCYL!FTVYFI
                                            :   :           .:*    **   *.* :  * . * **

CR4ARF T. brucei           CFVIVVCFFLLFWVVIFYWCFDCIVYFFCDVIILFIFFFYFVLCFLYCCFYLVCLVFFC-
CR4ARF T. vivax            CFVIVVCFFFLFWVVIFC!FIDCIVCFFCDVIILFIFFFCFVLCFLFCCFFLVCLVFFC-
CR4ARF L. tarentolae 13U   YIAFVCLFVLVCYVVILLLCFDCIVFFLFTVIIIFFFLFIFWVFIFLLLFWFVFGFLFFF
CR4ARF L. amazonensis      CIALFVCLFYVCYVVILLLCFDCIVFFLFIDIILFFFFFFYGLCLFF!LFLYLDLVFYFF
                            :.:.  :. : :***:   :**** *:   **:*:*:* : : ::   *  :  .::

CR4ARF T. brucei           IFCCVLCYFLIYFLCCFLFWVVFLFLFFFFVYVCFYLWLLLFC!FGCCCYLCIG------
CR4ARF T. vivax            IFCYVLCYFLICFLCCFLYWVVFLFLFFFFVYVYCFL!LLLFY!FGCCCYLCIG------
CR4ARF L. tarentolae 13U   FYVCVLIFYF------------------------EFLFMLFFVFCGFLLFVMFILFFISF
CR4ARF L. amazonensis      F!FCVWFFILNFCLCYFLFFADFCCLWCLFYFLCHFLF!LFYYFVGCYLYLYFV------
                           :   *  : :                         :*  *::   *   :: :

CR4ARF T. brucei           --LFICVFYFR!LWYWFYKMFF---------------
CR4ARF T. vivax            --LFICVLCF---------------------------
CR4ARF L. tarentolae 13U   FVLIVLVFCWLLFIFV-FRFFCMTFCILILIGLF!NL
CR4ARF L. amazonensis      --LFVCVLCFK---VG-YEFIFI--------------
                             *:: *: :
```

CR4 ARF Alignment with L. tarentolae 12U translation (Hypothetical Sequence)

```
CR4ARF T. brucei           -----IYCYLCVFIIILF!FWLCIFFFFIWCVCVLCTVYGIFYCCFVFCFCCLFVWVCFI
CR4ARF T. vivax            -----------------FFCLLCIFFFFIWCVCVLCIVCGIFCCCFFFCFFCLCVWVCFI
CR4ARF L. tarentolae 12U   QIH!NTYMYNCKSVV-VFGFFMY---------YFVC---CIFYFCFFFCLFDLCVMVYFI
CR4ARF L. amazonensis      ------DIKNIK!VI-FYYFYIFL---FTFVGWFLCIVVGIFWFYFFFYFCYL!FTVYFI
                                            :   :           .:*    **   *.* :  * . * **

CR4ARF T. brucei           CFVIVVCFFLLFWVVIFYWCFDCIVYFFCDVIILFIFFFYFVLCFLYCCFYLVCLVFFCI
CR4ARF T. vivax            CFVIVVCFFFLFWVVIFC!FIDCIVCFFCDVIILFIFFFCFVLCFLFCCFFLVCLVFFCI
CR4ARF L. tarentolae 12U   YIAFVCLFVLVCYVVILLLCFDCIVFFLFTVIIIFFFLFIFWVFIFLLLFWFVFGFLFFF
CR4ARF L. amazonensis      CIALFVCLFYVCYVVILLLCFDCIVFFLFIDIILFFFFFFYGLCLFF!LFLYLDLVFYFF
                            :.:.  :. : :***:   :**** *:   **:*:*:* : : ::   *  :  .:: :

CR4ARF T. brucei           FCCVL-CYFLIYFLCCFLFWVVFLFLFFFFVYVCFYLWLLLFC!FGCCCYLCIGLFICVF
CR4ARF T. vivax            FCYVL-CYFLICFLCCFLYWVVFLFLFFFFVYVYCFL!LLLFY!FGCCCYLCIGLFICVL
CR4ARF L. tarentolae 12U   FMFVFWFFILNFCLCCFLFFVDFCCLWCLFYFLYHFLF!LCWCFVGCYLYLCFDFFVWRF
CR4ARF L. amazonensis      F!FCVWFFILNFCLCYFLFFADFCCLWCLFYFLCHFLF!LFYYFVGCYLYLYFVLFVCVL
                           *   .  ::*   ** **::. *  *: :* ::  :*  * :  .**  ** : :*:  :

CR4ARF T. brucei           YFR!LWYWFYKMFF
CR4ARF T. vivax            CF------------
CR4ARF L. tarentolae 12U   VF!F!LDYFKI---
CR4ARF L. amazonensis      CFKVGYEFIFI---
                            *
```

C. ND3

ND3 ORF Alignment

```
ND3ORF T. brucei        --LLSLFLYLFLILWFIILFIGFFLCFLCFLLHFFDCFRCCLWFSCGLYDMNSRLVFYTL
ND3ORF T. vivax         --MLSIFLYLVLCLWFIILLLGFFLCFLCFLLHFFDCFRCCLWFSCGLYDMNSRLVFYTL
ND3ORF L. tarentolae    ------------------------FGRREKVLHFFDCFRCCLWFSCGLYDMNSRFVYVSI
ND3ORF L. amazonensis   MFFCSFYFNFVLIFCMLILSIGVLFYIFMFLLHFFDCFRCCLWFSCGLYDMNSRLCYIFI
                                                :     :***********************: :  :

ND3ORF T. brucei        DLCFVSCLFFVLLNSIICVLLFVFVIVLFYFCYGFLFLWFLFFVVCIGFVWYFWDHVYLC
ND3ORF T. vivax         DLCFVSCLFFVLLNSVICVLLFVFVLVLFYFCYGFLFLWFLFFVLLLGFVWYFWDHVY--
ND3ORF L. tarentolae    DLCFAVLLCFVMFYSIIGLILFLIVVVLYFMCKL-FFVWFCFVFLL------FWSIV---
ND3ORF L. amazonensis   DLCFAILLCFVLFYSNFGLIIFVLVVLLYFMCKL-FFVWFLLLFFM------LWLMYLI-
                        ****.  * **:: * : :::*::*::*:::*   :*:** :...       :*

ND3ORF T. brucei        GVILFCLWCFLLYYTYYINKYIK----------
ND3ORF T. vivax         ---------------------------------
ND3ORF L. tarentolae    FNIWFCV---LFVFIFFLLILINSVFSYLILK!
ND3ORF L. amazonensis   FDSVYCL---FLFFFY!----------------
```

ND3 ARF Alignment

```
ND3ARF T. brucei        -SKNPRLFTLVCYHYFYICF-W-YCGLLFYL!VFFYVFYVFYCIFLIVFVVVCGFRVVCM
ND3ARF T. vivax         -----------CYQFFCIWF-C-VCGLLFYC!VFFYVFYVFCYIFLIVFVVVYDFHVVCM
ND3ARF L. tarentolae    HSKNSSI-Y-----------------NLYRSIGISLGGGKKCCIFLIVFVVVYDFRVVCM
ND3ARF L. amazonensis   TQKNSRFKFKICFFVHFILILC!FFVCWF!VLVYYFIFLCFCCIFLIVFVVVYDFHVVCM
                                                    :              ********* .*:****

ND3ARF T. brucei        IWIHVWCFIHWIYVLLVVCFLYC!IPLFVFCCLFLW!CCFIFVMVFCFCGFCFLLYV!DL
ND3ARF T. vivax         IWIHVWCFIH!IYVLLVVCFLCYWILLFVFCYLYLCWYCFIFVMVFYFCGFCFLCCY-GL
ND3ARF L. tarentolae    TWIHDLFMFL!IYVSQFYYVLLCFIPLLV!FYF!!LWCCILCVNC-FLCGFVLYFCYFGV
ND3ARF L. amazonensis   TWIHVYVIFL!IYVSQFYCVLYCFIPILVWLFLY!WCCYILCVNY-FLCDFYCYFLCYGW
                         ***   ::  ***  .  .*   * ::*   :      :: *    :*.*       .

ND3ARF T. brucei        CGIFGITYI--CVV!FYFVYDVFCCIIHIILINI!---
ND3ARF T. vivax         CGIFEIMYI-----------------------------
ND3ARF L. tarentolae    L--YLIFDSVYCLFLFFF-Y!F!!IVYSLI!Y!NNK!I
ND3ARF L. amazonensis   CI!YLILCTV-CFC-FFF-INLINSVK--LSLD!NN--
                           : *
```

D. ND7 5' Editing Domain

ND7 5' Editing Domain ORF Alignment

```
ND7ORF T. brucei          -----------MLFLVVFLHLYRFTFGPQHPAAHGVLCCLLYFCGEFIVYIDCIIGYLHR
ND7ORF T. vivax           ----------MFIFVVVFLHLYRFTFGPQHPAAHGVLCCLLYFCGEFIVYIDCIIGYLHR
ND7ORF L. tarentolae      ILFSRLHDNYILYLLIVFLHLYRFTFGPQHPAAHGVLCCLLYLSGEFITYIDVIIGYLHR
ND7ORF P. serpens         -------IIFIFFIFVVFLHLYRFTFGPQHPAAHGVLCCLLYFSGEYITYIDVIIGYLHR
                                     : :.:**************************:.**:*.*** *******

ND7ORF T. brucei          GTEKLCE
ND7ORF T. vivax           GTEKLCE
ND7ORF L. tarentolae      GTEKLCE
ND7ORF P. serpens         GTEKLCE
                          *******
```

ND7 5' Editing Domain ARF Alignment

```
ND7ARF T. brucei          -IQKNMTTWYSIIVIFGSFFTFVSFYIWSTASRSTWCFMLFIVFLWWIYCLYWLYYRLFA
ND7ARF T. vivax           ------------VYFCCSFFAFVSFYIWPTASRSTWCFMLFIVFLWWIYCLYWLYYRLFA
ND7ARF L. tarentolae      LNFI!PTTR!LYFIFINCFFTLV!IYFRTPASSSPWRIMLFIISFWRIYNVYRCNYWVFT
ND7ARF P. serpens         -------SNNFYFFYFCCFFAFVSFYVWSTASSRTWCVMLFVIFFRWVYNIYWRNYRLLT
                                      .    .**::* :*.   **   * .***:: :  :* :*   * :::

ND7ARF T. brucei          SWYRKVMWI!
ND7ARF T. vivax           SRYRKVMWV!
ND7ARF L. tarentolae      SRYRKVMWI!
ND7ARF P. serpens         PWNGKIVWI!
                              *::*:*
```

E. ND9

ND9 ORF Alignment

```
ND9ORF T. brucei         MCIFLCLFRLCFCLILFYCLCCRWCFVCFVDCSFLFFYCFVSFFLFYCMFLFFNLWFLFL
ND9ORF T. vivax          MGLLLCLFRLCFCLILFYCLCCRWCFVCFVDCSFLFFYCFVSFFLFYCMFLFFNLWFLFL
ND9ORF L. tarentolae     MFLFLIMFRCVFVLLLFFCLCCRWVFLCFVDCSFVFFYLFVCFFLFFVMFLFFNLWFFLL
ND9ORF L. amazonensis    MFLFLIMFRCVFVLCLFFCLCCRWVFLCFVDCSFVFFYLFVCFFLFFVMFLFFNLWFFLL
                         * ::* :**  * * **:****** *:*******:*** **.****: *********::*

ND9ORF T. brucei         YCCDLLLIDFCGFCFCRFMLLYILFCLFLCVRLCFVLCCLFVFFGLCFSFSCFCYAFLLL
ND9ORF T. vivax          YCCDLLLIDFCGFCFCRFMLLYILFCLFLCFRLCFVLCCLFLFFGLCFSFSCFCYAFLLL
ND9ORF L. tarentolae     YCLDLFCIDFCGFCFVRFILIYVLFCLLLCFRVSFVLICFFLFFGLVFSLFFCSYALCIF
ND9ORF L. amazonensis    YCLDLFCIDFCGFCFVRFVLLYVLFCLILCFRVSFVLICFFLFFGLVFSLFFCSYALCIF
                         ** **: ******** **:*:*:****:**.*:.*** *:*:**** **:   .**: ::

ND9ORF T. brucei         ERECFDLFGFYFVGNDILHRLFVDWFFVGFFLLKCYPLFGLFVLLFCVLVEEIVCTFTML
ND9ORF T. vivax          ERECFDLFGFYFVGNDVLHRLFVDWFFVGFFLLKCYPLFGLFVLLFCVLVNEMICTFTMV
ND9ORF L. tarentolae     EREVFDLFGFVFCGNDCLHRFYVDWFFVGFFLCKVYPLFGLFMLNFCMLCEDIVVIATSC
ND9ORF L. amazonensis    ERECFDLFGFVFCGNDCLHRFYVDWFFVGFFLCKVYPLFGLFVLNFCMLVEEIIVYATCC
                         *** ****** * *** ***::********** * *******:* **:* ::::   *

ND9ORF T. brucei         FLLLHTNFYLHYFI!---------
ND9ORF T. vivax          FLLLHTNFYLHFFV!---------
ND9ORF L. tarentolae     FVLCFSNFAI!-------------
ND9ORF L. amazonensis    FVLVFPILHLFNLIYDNADLNIN!
                         *:* .  : :
```

ND9 ARF Alignment

```
ND9ARF T. brucei           NINLIFFINIILCVYFYVYFVYVFV!FYFIVCVVDGVLFVLLIVVFCFFIVLLVFFCFIV
ND9ARF T. vivax            ------------WVCCCVYFVCVFVWFCFIVCVVGGVLFVLLIVVFCFFIVLLVFFCFIV
ND9ARF L. tarentolae       -RYII!YLLSIICFYFWLCFVVCLCCCYFFVCVVDGFFYVLLIVVLFFFICLCVFFYFLW
ND9ARF L. amazonensis      IINCLY!NLIKLCFYF!LCFVVYLYYVYFFVYVVGEFFYVLLIVVLFFFICLCVFFYFLW
                                        .   : **  :    *:* **. .::******: *** * *** *:

ND9ARF T. brucei           CFYFLICDFCFYIVVICYWLIFVVFVFVVLCCCIFYFVCFCVFVCVLFCVVCLYFLDCVL
ND9ARF T. vivax            CFYFLIYGFCFCIVVIYCWLIFVVFVFVVLCYYIFCFVCFCVFVYVLFCVVCFCFLDCVL
ND9ARF L. tarentolae       CFYFLIYDFFYCIV!IYFV!IFAVFVLFVLFWYMFCFVYYYYVFE!VLYWFVFFCFLVWFL
ND9ARF L. amazonensis      CFCFLIYDFFYCIVWICFV!IFAVFVLFDLFYYMFCFV!FCVFG!VLYWFVFFYFLVWFL
                           ** *** .* : ** *    **.***:. *   :* ** : **  **: .* : **  .*

ND9ARF T. brucei           VLVVFVMRFCCWNANVLICLVFILLVMIFYIVYLLIGFLLVFFCWSVIHYLVCLYCYFVC
ND9ARF T. vivax            VLVVFVMRFYC!NVSVLICLVFILLVMMFYTVYLLIDFLLVFFCWSVIHYLVCLYYCFVF
ND9ARF L. tarentolae       VYFFVVMRYVFLNVKFLICLVLFFVVMIVYIVFMLIDFLLVFFCVKFIHCLVCLCWIFVC
ND9ARF L. amazonensis      VYFFVVMRYVFLSENVLICLVLFFVVMIVYIVFMLIDFLLVFFYVKFIHCLVYLCWIFVC
                           * ...***:   . ..*****::::**:.* *::**.******  ..** ** *   **

ND9ARF T. brucei           WWRR!YVRLQCYFCCCIPIFICIILFNITVVILNFSL----
ND9ARF T. vivax            !LMRWYVRLQWFFCCCIPIFICIFLF---------------
ND9ARF L. tarentolae       YVKILLWLPLVVLCCVFPILQYSFYFIFV---MNLSIKLIY
ND9ARF L. amazonensis      WLKRLLFMLHVVLC!FFQFCIYLI!FMIM---LT!TLTKF-
                                       :*   : :    : *
```

F. RPS12

RPS12 ORF Alignment

```
RPS12ORF T. brucei          ----------MWFLYGCCLRFVLFVLCYYMSPRLPSSGNRRVLYAVFYLYNFVWMLRCFF
RPS12ORF T. vivax           ----------MWFLYGCCLRFVLFVLCYYMSPRLPSSGNRRVLYAVFYLYNFVWLLRCFF
RPS12ORF L. tarentolae      --------MRVLFLYGLCVRFLYFCLVLYLSPRLPSSGNRRCLYAICYMFNILWFFCVF-
RPS12ORF L. amazonensis     TFLNLIYFVRVLYLYGLCVRFLFLCLVLYLSPRLPSSGNRRCLYAISIMFNILWYFLVF-
RPS12ORF P. serpens         -----MFFVRSYCLYGFCVRFCFVFLCIYVSPRLPSSGNRRVYVVCFNLYSFVIYCFLFG
RPS12ORF Perkinsela         ------------MLFGFLVRYGFIEFFFFVSPRLPSSGNRFCYELDMRFFFVCYDFVLLG
                                         *:*  :*:  . :  ::**********        :: .      :

RPS12ORF T. brucei          CC-FIGLVMSLFIIEGGGF---VDLP-GVKYYTRIVS!--
RPS12ORF T. vivax           CCVFFGLHLSLFIIEGGGF---VDLP-GIKYYTRMFIN!-
RPS12ORF L. tarentolae      CCVCF-LNHLLFIVEGGGF---IDLP-GVKYFSRFFLNA!
RPS12ORF L. amazonensis     CCFVF-VIFQLFIVEGGGF---IDLP-GVKYFSRFCNVS!
RPS12ORF P. serpens         CCVICYSQSFYFLCEGGGF---VDLP-CIKLYVRVPIA!-
RPS12ORF Perkinsela         FSV---LLSSLVFYEGFGFWLFMDVPFGLYYFSRG!----
                             .         .: ** **   :*:*  :  : *
```

RPS12 ARF Alignment

```
RPS12ARF T. brucei          NTLLITN!SK--------YILFFLRMWFCMVVVYVLFYLFYVIIWVRDCPVPVTDVYCMP
RPS12ARF T. vivax           -------------------------CDFCMVVVCVLFCLFYVIIWVPDCPVPVIDVCCMP
RPS12ARF L. tarentolae      ---NTYRPI!--------IIFILCVYYFCMVYVFVFYIFVWFYI!VHDYLVPVIDVVYMQ
RPS12ARF L. amazonensis     HK-YLFRPF!--------I!FILFVFYICMVYVFVFYFYVWFYI!VHDYQAPVIDVVYMQ
RPS12ARF P. serpens         -----LKPIF!LS!YFYLYLCFLFVVIVYMVFVYVFVLYFYVYMLVPVYPVQVIVVFMLF
RPS12ARF Perkinsela         ---------------------------CCLVFWFVMVL!SFFFLLALVCPVLVIGFVMSW
                                                         :*   *:    :. : .    . *  .

RPS12ARF T. brucei          YF--IYIILFGCCVVFFVVL-LV!LCHYLL!RVVVLLIYPV!SIIHVL!VRFRYKICF--
RPS12ARF T. vivax           CF--ICIILCGCCVVFFVVCFLVCICRYLL!RV-VLLIYPV!SIIHVCLLI---------
RPS12ARF L. tarentolae      YV--ICLIFYDFFVFFVV-FVFWIIC-CL!LKVVVLLICQE!SIFHVFFWMRKQ!VIIKI
RPS12ARF L. amazonensis     LV--LCLIFYDIFWFFAV-LFLWFFS-CL!LKVVVLLICQE!SIFRVFVMCRKFNYLYFY
RPS12ARF P. serpens         VL--ICIVLLFIVFYLVVVLFVILRVFIFYVRVVVLLIYHV!SYMSVCQ!PK!IIAS---
RPS12ARF Perkinsela         IWGFFLFVMILCCWVFPFCCQVWFFMKVLV---------------FGCLWMYRLDCIIFP
                                : :::      : .   .      :

RPS12ARF T. brucei          ----
RPS12ARF T. vivax           ----
RPS12ARF L. tarentolae      ILFR
RPS12ARF L. amazonensis     KN--
RPS12ARF P. serpens         ----
RPS12ARF Perkinsela         VV--
```

G. ND8 (Nondual-coding)

ND8 ORF Alignment

```
ND8ORF T. brucei        MFFFDFLFFFFVCFYMCFVCCVTICLPIELTIVSLLVRGNHFLRFYWCGLERCIACRLCD
ND8ORF T. vivax         MFFFDFLFFFFVCFYMCFVCCVTICLPIELTFCSLLTRGNHFLRFYWCGLERCIACRLCD
ND8ORF L. tarentolae    MFVYDFCFSFFVCFYMCFLCCVTLVLPLELTIVSICVRGNHFLRFYWCGLERCIACRLCD
ND8ORF L. amazonensis   MFCYDFVFSFFVCFYMCFLCCVTLILPCEITIVSICARGHHFLRFYWCGLERCIACRLCD
ND8ORF P. serpens       MFFFDFFFCFFCFVYMCFCCCVTIVVPCEVSLCSFLVRGTHFLRFYWCGLERCIACRMCD
                        ** :** * **  .**** ****: :* *::: *: .** *****************:**

ND8ORF T. brucei        LICPSLALDVRVGWSFGGHRFADWFTLSYRRCIYCGFCMHVCPTDAITHSLFVMCFCCLA
ND8ORF T. vivax         LICPSLALDVRVGWSFGGHRFADWFTLSYRRCIYCGFCMHVCPTDAITHSLFVMCFCCLA
ND8ORF L. tarentolae    FICPSLALDVRCVRSLCGYRFSDVFNISYRRCIYCGFCMHVCPTDAITHSCFLLFCCCIA
ND8ORF L. amazonensis   FICPSLAIDVRCIRSLCGYRYSDLFYISYRRCIYCGFCMHVCPTDAITHSCFLLFCCCIA
ND8ORF P. serpens       YICPSVAIDVRCGVSLIGHRFAHLFFISYRRCIYCGFCMHVCPTDAITHSFVVLFSVLLS
                         ****:*:***   *: *:*::. * :*********************** .::    ::

ND8ORF T. brucei        MYLLAPKFLLFGCCFMLFDFYLCFV!
ND8ORF T. vivax         MYLLAPKFLLFGCCFMLFDFYLCFV!
ND8ORF L. tarentolae    MYLCAPKFVLFGCCFMLFDFYLCFV!
ND8ORF L. amazonensis   MYLCAPKFVLFGCCFMLFDFYLCFV!
ND8ORF P. serpens       SYLVAPKFILFGCCFMVFDLFLCFC!
                         ** ****:*******:**::***
```

ND8 ARF Alignment

```
ND8RF2 T. brucei        NLIILSFGWLLFFYFFIFVCFFLIFCFFFLFVFICVLFVVLLFVYPLS!PLLVYWFVVTI
ND8RF2 T. vivax         -------------------CFFLIFCFFFLFVFICVLFVVLPFVYPLN!HFVVCWPVVTI
ND8RF2 L. tarentolae    ---------KHI!CIRLKECLFMIFVFLFLFVFICVFYVVLLWFYHWSWPLLVFVFVVTI
ND8RF2 L. amazonensis   ---------NIIRSILIKICFVMILFFLFLFVFICVFYVVLLWFYHVRLPLLVFVLVVII
ND8RF2 P. serpens       ----------!I!CVNVIKCFFLIFFFVFFVLFICVFVVVLPLLFHVKYHCVVFWFVVLI
                                           *:.:*: *.*:.:****: ***  .:      :*   ** *

ND8RF2 T. brucei        FCVFIGVV!SVVLLVVYVI!FALV!HWMFVLGGVLVVIVLRIDLHWVIVVVFIVVFVCMF
ND8RF2 T. vivax         FCVFIDVV!NVVLLVVYVIWFVPV!H!MFVLGGVLVVIVLQIDLHWVTDVVFIVVFVCMF
ND8RF2 L. tarentolae    FCVFIDVV!NVVLPAVYVILYAQV!L!MFVVLEVYVVIGFPMCLILVIVVVFIVVFVCMF
ND8RF2 L. amazonensis   FCVFIDVV!NGVLPAVYVILYALVWPLMFVVLEVYVVIVIPIYFILVIVVVFIVVFVCMF
ND8RF2 P. serpens       FYVFIGVV!NVVLLVVCVIIFVLVLP!MFVVVLVWLVIVLHICFLLVIDVVFIVVFVCMF
                        * ***.***. ** .* ** :. *   ***:  * :** : : :  *  ***********

ND8RF2 T. brucei        ARQMPLRIHCLLCVFVV!PCIYWRPSFYCLVVVLCYLIFICVLCSYLFWVI------YCV
ND8RF2 T. vivax         VQQMPLHIHCLLCVFVV!PCIYWRPSFCYLVVVLCCLIFICVLC----------------
ND8RF2 L. tarentolae    VQQTPLRIHVFCYFVVVLPCIYAHLNLFYLVVVLCYLIFICVLFSLVYLEKY-IWLII!!
ND8RF2 L. amazonensis   VPPMLLRIHVFCYFVVVLPCIYAHLNLFYLVVVLCCLIFICVLFNL!FEYFFYIFYVVCS
ND8RF2 P. serpens       VQPMQLPIHLLFYLVCCYPVIWLHPNLFCLVVVLWFLIYFCVFVSCIV!FI!----LFVV
                        .    * ** :  ..   * *: : .:  *****  **::**:

ND8RF2 T. brucei        YDLKKFTVKLNFD!
ND8RF2 T. vivax         --------------
ND8RF2 L. tarentolae    INF-----------
ND8RF2 L. amazonensis   KNLVNV--------
ND8RF2 P. serpens       !!!!K!RTK!Y!--
```
