# Supplementary material for: Mitochondrial dual-coding genes in Trypanosoma brucei
Source: PLoS Negl Trop Dis. 2017 Oct 9;11(10):e0005989. doi: 10.1371/journal.pntd.0005989 (PMC5650466; doi:10.1371/journal.pntd.0005989)
Supplement: S1 Table — A—C: Major CR3 mRNA and gRNA sequence classes. The CR3 mRNA transcriptome was generated using the TREU667 cell line. Identified sequences were then used to search gRNA transcriptomes from four different cell lines: EATRO 164 Bloodstream (BS), EATRO 164 procyclic (PC), TREU 927 procyclic and TREU 667 procyclic. ORF = previously identified Open Reading Frame (purple protein sequence). ARF = Newly identified Alternative Reading Frame (green protein sequence). Alternatively edited nucleotides are shown in Red. Inserted U-residues are lowercase while deleted U-residues are shown as asterisks. Canonical Watson-Crick base pairs (|); G:U base pairs (:). Previously identified start codons are doubled underlined. Potential upstream AUG start codons are indicated by wave underlines. gRNAs were sorted based on guiding sequence class. Sequence variations observed in the 3’-U-tail were ignored in assigning class. Transcript copy number (Reads), were determined by adding all gRNAs of the same sequence class. Only major sequence classes are shown (defined as containing greater than 100 transcript copies). In the case of rare transcripts, the identified gRNA are shown regardless of copy number. gRNA transcript numbers varied greatly between the different cell lines. Interestingly, the most abundant mRNA (CR3 Form C, 7147 reads) had the fewest identified gRNA reads. (PDF) [file pntd.0005989.s003.pdf]

S1 Table: Identified CR3 mRNA and gRNA transcripts

<table>
<tr><th colspan="2">A. CR3 Form A — mRNA Sequence</th><th>Reads</th></tr>
<tr><td colspan="2">AUGUGUAUGAUAUAUAAuuAAuuAuuuuCAuuuuAuGuuuGA****UUGuuuGGuuuuGuuGuuuuUUUAuuGuuuGuuuGuA</td><td>2114</td></tr>
<tr><td colspan="2">AUGUGUAUGAUAUAUAA--AAuuAuuuuCAuuuuAuGuuuGA****UUGuuuGGuuuuGuuGuuuuUUUAuuGuuuGuuuGuA</td><td>1217</td></tr>
<tr><td colspan="2">AUGUGUAUGAUAUAUAA--AA--AuuuuCAuuuuAuGuuuGA****UUGuuuGGuuuuGuuGuuuuUUUAuuGuuuGuuuGuA</td><td>480</td></tr>
<tr><th colspan="2">Fully Edited Form</th><th>Reading Frame</th></tr>
<tr><td colspan="2">M C M I Y N ST M F D C L V L L F F Y C L F V
AUGUGUAUGAUAUAUAAuuAAuuAuuuuCAuuuuAuGuuuGA****UUGuuuGGuuuuGuuGuuuuUUUAuuGuuuGuuuGuA
|||||||:|:|||||:|:|||:|:||| |||:|:|||||||||||
NUUAAUUAGUGAAAGUGAGAUAUAGACU----AACGAGCCAAAACAACAUGUAUA</td><td>ORF</td></tr>
<tr><th>Cell Line</th><th>gRNA Sequence</th><th>Reads</th></tr>
<tr><td>EATRO 164 PC</td><td>ATATGTACAACAAAACCGAGCAATCAGATATAGAGTGAAAGTGATTAAT$_N$</td><td>21297</td></tr>
<tr><td>TREU 927 PC</td><td>ATATAACAACAAAACTGAACAATCAAATGTAGAGTGAAAGTGATTAAT$_N$</td><td>3398</td></tr>
<tr><td>TREU 667 PC</td><td>ATATAACAACAAAACTGAACAATCAAATGTAGAGTGAAAGTGATTAAT$_N$</td><td>452</td></tr>
</table>

<table>
<tr><th colspan="2">B. CR3 Form B — mRNA Sequence</th><th>Reads</th></tr>
<tr><td colspan="2">AUGUGUAUGAUAUAUAuuAuuAuuuAuuuAuuuuCAuuAuGuuuGA****UUGuuuGGuuuuGuuGuuuuUUUAuuGuuuGuuuG</td><td>413</td></tr>
<tr><td colspan="2">AUGUGUAUGAUAUAUA--AuuAuuuAuuuAuuuuCAuuAuGuuuGA****UUGuuuGGuuuuGuuGuuuuUUUAuuGuuuGuuuG</td><td>460</td></tr>
<tr><th colspan="2">Fully Edited Form</th><th>Reading Frame</th></tr>
<tr><td colspan="2">M C M I Y I I I Y L F S L C L I V W F C C F F I V C L
AUGUGUAUGAUAUAUAuuAuuAuuuAuuuAuuuuCAuuAuGuuuGA****UUGuuuGGuuuuGuuGuuuuUUUAuuGuuuGuuuG
|||:|||:|||:|:|:|::|:||||||:|::|| ||||||||||
NUAUGAUAGUAAGUGAGUGGAGGUAAUAUAGGCU----AACAAACCAAUAUAUA</td><td>ARF +1</td></tr>
<tr><th>Cell Line</th><th>gRNA Sequence</th><th>Reads</th></tr>
<tr><td>EATRO 164 PC</td><td>ATATATAACCAAACAATCGGATATAATGGAGGTGAGTGAATGATAGTAT$_N$</td><td>5</td></tr>
<tr><td>TREU 927 PC</td><td>ATATATAACCAAACAATCGGATATAATGGAGGTGAGTGAATGATAGTAT$_N$</td><td>14164</td></tr>
<tr><td>TREU 927 PC</td><td>ATATATAACCAAACAATCGGATATAATGGAGGTGAGTGAATGATAGTATAT$_N$</td><td>642</td></tr>
<tr><td>TREU 927 PC</td><td>ATATATAACCAAACAATCGGATATAATGGAGGTGAGTAAATGATAGTAT$_N$</td><td>114</td></tr>
<tr><td>TREU 667 PC</td><td>ATATATAACCAAACAATCGGATATAATGGAGGTGAGTGAATGATAGTAT$_N$</td><td>1936</td></tr>
<tr><td>TREU 667 PC</td><td>TATATAACCAAACAATCGGATATAATGGAGGTGAGTGAATGATAGTATAT$_N$</td><td>236</td></tr>
</table>

<table>
<tr><th colspan="2">C. CR3 Form C — mRNA Sequence</th><th>Reads</th></tr>
<tr><td colspan="2">AUGUGUAUGAUAUAUAAAAACA--AuGuGuA*****UGuuGuuGuuuuGuuuuG***AuuuuGGuuGuACAuuuuuuuuG</td><td>7147</td></tr>
<tr><td colspan="2">AUGUGUAUGAUAUAUAAAAACA--A-GuGuA*****UGuuGuuGuuuuGuuuuG***AuuuuGGuuGuACAuuuuuuuuG</td><td>889</td></tr>
<tr><td colspan="2">AUGUGUAUGAUAUAUAAAAACAuuA-GuGuA*****UGuuGuuGuuuuGuuuuG***AuuuuGGuuGuACAuuuuuuuuG</td><td>565*</td></tr>
<tr><td colspan="2">AUGUGUAUGAUAUAUAAAAACA-uAuGuGuA*****UGuuGuuGuuuuGuuuuG***AuuuuGGuuGuACAuuuuuuuuG</td><td>505</td></tr>
<tr><th colspan="2">Fully Edited Form</th><th>Reading Frame</th></tr>
<tr><td colspan="2">M C M I Y K N N V Y V V V L F W F W L Y I F F V
AUGUGUAUGAUAUAUAAAAACAAuGuGuA*****UGuuGuuGuuuuGuuuuG***AuuuuGGuuGuACAuuuuuuuuG
||||:|:|| |:|:::|:||:|:::||: |:||:||:|||#|||||
NUUAUAUAU-----AUAGUGAUAAGAUGGAAU---UGAAGCCGACACGUAAAUUAAUAUA</td><td>ARF +1</td></tr>
<tr><th>Cell Line</th><th>gRNA Sequence</th><th>Reads</th></tr>
<tr><td>EATRO 164 PC</td><td>ATAATAAAAATGCACAACTAGAATTGAAGTAAAGTGATGATATATAT$_N$</td><td>122</td></tr>
<tr><td>EATRO 164 PC</td><td>AAAATGCACAACTAGAATTGAAGTAAAGTGATGATATATATAT$_N$</td><td>3</td></tr>
<tr><td>EATRO 164 PC</td><td>ATATATAAAATGTACAACCAGAATTAAGATAAAGTGATGATGTATATAT$_N$</td><td>1</td></tr>
<tr><td>EATRO 164 BS</td><td>ATAATAAAAATGCACAACTAGAATTGAAGTAAAATGATGATATATAT$_N$</td><td>453</td></tr>
<tr><td>EATRO 164 BS</td><td>ATATTAAAAATGCACAACTAGAATTGAAATAAAGTGATGGTATATAT$_N$</td><td>224</td></tr>
<tr><td>EATRO 164 BS</td><td>ATAATAAAAATGCACAACTAGAATTGAAGTAAAATGATGATATATATAT$_N$</td><td>2</td></tr>
<tr><td>TREU 927 PC</td><td>ATAATAAAAATGCACAACTAGAATTGAAATAAAGTGATGGTATATAT$_N$</td><td>166</td></tr>
<tr><td>TREU 927 PC</td><td>ATATAATTAAATGCACAGCCGAAGTTAAGGTAGAATAGTGATATATATAT$_N$</td><td>117</td></tr>
<tr><td>TREU 927 PC</td><td>ATATATAAAATGTACAACCAGAATTAAGATAAAGTGATGATGTATATAT$_N$</td><td>86</td></tr>
<tr><td>TREU 667 PC</td><td>ATAATAAAAATGCACAACTAGAATTGAAATAAAGTGATGGTATATAT$_N$</td><td>53</td></tr>
<tr><td>TREU 667 PC</td><td>ATATAATTAAATGCACAGCCGAAGTTAAGGTAGAATAGTGATATATAT$_N$</td><td>15</td></tr>
<tr><td>TREU 667 PC</td><td>ATATATAAAATGTACAACCAGAATTAAGATAAAGTGATGATGTATATAT$_N$</td><td>14</td></tr>
<tr><td>TREU 667 PC</td><td>TATAATTAAATGCACAGCCGAAGTTAAGGTAGAATAGTGATATATATAT$_N$</td><td>5</td></tr>
</table>

* no gRNAs identified.
