# Supplementary material for: Mitochondrial dual-coding genes in Trypanosoma brucei
Source: PLoS Negl Trop Dis. 2017 Oct 9;11(10):e0005989. doi: 10.1371/journal.pntd.0005989 (PMC5650466; doi:10.1371/journal.pntd.0005989)
Supplement: S2 Table — A—J: ND7 gRNA major classes and predicted editing patterns. ND7 terminal (5’ most) gRNA populations and the predicted mRNA sequence generated. Predicted sequences presented are based on the most abundant gRNAs that generate each reading frame found in the four gRNA transcriptome databases. Initial characterization of the ND7 transcript was done using the EATRO 164 cell line and is unusual in that it is edited in two distinct domains [20]. While the 5’ domain was edited in both life cycle stages, complete editing of the 3’ domain was only detected in bloodstream stage parasites. Interestingly, the most abundant EATRO 164 PC (procyclic or insect form) gRNA would generate a sequence that brings the 5’ most AUG into a +2 frame. The ARF is 65 AA long and involves the entire 5’ editing domain. In contrast, the most abundant gRNAs in the EATRO 164 Bloodstream stage library (EATRO 164 BS), would generate sequences that use the originally described ND7 ORF). While gRNA transcript numbers again varied greatly between the different cell lines, all three cells lines had gRNA sequence variants that allowed access to both reading frames. (PDF) [file pntd.0005989.s004.pdf]

S2 Table: ND7 5’ most gRNA populations and predicted mRNA sequences generated.

<table>
<tr><th>A. ND7 Form A</th><th>Predicted mRNA Sequence</th><th>Reading Frame</th></tr>
<tr><td colspan="2"><pre>
          M I S I I L C Y F W ST
M T T W ST   M L F L V V F L H L Y R F T F G P Q
AUGACUACAUGAUAAGUAuCAuuuuAuGuuAuuuuuGGuAGuuuuuuuACAuuuGuAuCGuuuuACAuuuG*GUCCACAG
                :|||||||:|||:|||:|||:||:||||:|:|||||||||||||
               $_N$UAUAGUAAGAUGCAAUGAAAGCCGUCAAGAGAAUGUAAACAUAUAAA
</pre></td><td>ORF</td></tr>
<tr><td>Cell Line</td><td>gRNA Sequence</td><td>Reads</td></tr>
<tr><td>EATRO 164 BS</td><td>AAATATACAAATGTAAGAGAACTGCCGAAAGTAACGTAGAATGATAT$_N$</td><td>20487</td></tr>
<tr><td>TREU 927 PC</td><td>AAATATACAAATGTAAGAAAACTATCGAGAGTGATGTAGAATGATAT$_N$</td><td>10537</td></tr>
<tr><td>TREU 667 PC</td><td>AAATATACAAATGTAAGAAAACTATCGAGAGTGATGTAGAATGATAT$_N$</td><td>787</td></tr>
</table>

<table>
<tr><th>B. ND7 Form B</th><th>Predicted mRNA Sequence</th><th>Reading Frame</th></tr>
<tr><td colspan="2"><pre>
M T T W Y S I I L C Y F W ST
          M I ST   M L F L V V F L H L Y R F T F G P Q
AUGACUACAUGAUAuAGUAuCAuuuuAuGuuAuuuuuGGuAGuuuuuuuACAuuuGuAuCGuuuuACAuuuG*GUCCACAG
              |||:|||||||:||:||||:|||:||:||||:|:|||||||||||||
             $_N$UAUUAUAGUAAGAUGCAAUGAAAGCCGUCAAGAGAAUGUAAACAUAUAAA
</pre></td><td>ORF</td></tr>
<tr><td>Cell Line</td><td>gRNA Sequence</td><td>Reads</td></tr>
<tr><td>EATRO 164 BS</td><td>AAATATACAAATGTAAGAGAACTGCCGAAAGTAACGTAGAATGATATTAT$_N$</td><td>35079</td></tr>
<tr><td>TREU 927 PC</td><td>AAATATACAAATGTAAGAAAACTATCGAGAGTGATGTAGAATGATATTAT$_N$</td><td>38432</td></tr>
<tr><td>TREU 667 PC</td><td>AAATATACAAATGTAAGAAAACTATCGAGAGTGATGTAGAATGATATTAT$_N$</td><td>4365</td></tr>
</table>

<table>
<tr><th>C. ND7 Form C</th><th>Predicted mRNA Sequence</th><th>Reading Frame</th></tr>
<tr><td colspan="2"><pre>
M T T W ST
          M I S T F M L F L V V F L H L Y R F T F G P Q
AUGACUACAUGAUAAGUACAuuuAuGuuAuuuuuGGuAGuuuuuuuACAuuuGuAuCGuuuuACAuuuG*GUCCACAG
                    |||||::|:|:||:|:::||::|:||||||||||||||||
                   $_N$UAAAUGUAGUGAAGAUUGUCGGAGAAAUGUAAACAUAGCAUAUACA
</pre></td><td>ORF</td></tr>
<tr><td>Cell Line</td><td>gRNA Sequence</td><td>Reads</td></tr>
<tr><td>TREU 927 PC</td><td>ACATATACGATACAAATGTAAAGAGGCTGTTAGAAGTGATGTAAAT$_N$</td><td>75654</td></tr>
</table>

<table>
<tr><th>D. ND7 Form D</th><th>Predicted mRNA Sequence</th><th>Reading Frame</th></tr>
<tr><td colspan="2"><pre>
M T T W ST
          M I I V S F M L F L V V F L H L Y R F T F G P Q
AUGACUACAUGAUAAuuGUAuCAuuuAuGuuAuuuuuGGuAGuuuuuuuACAuuuGuAuCGuuuuACAuuuG*GUCCACAG
               |||:||||||:||||:|||::|:||:|||:||:||||||||||||||
              $_N$UAAUAUAGUGAAUAUAAUGGAGACUAUCGAAGAAAUGUAAACAUAUAAA
</pre></td><td>ORF</td></tr>
<tr><td>Cell Line</td><td>gRNA Sequence</td><td>Reads</td></tr>
<tr><td>EATRO 164 PC</td><td>AAATATACAAATGTAAAGAAGCTATCAGAGGTAATATAAGTGATATAAT$_N$</td><td>240</td></tr>
<tr><td>TREU 927 PC</td><td>ATATACACAAATGTAAAGAGACTATCGAGAGTGACATAAGTGATATAAT$_N$</td><td>477</td></tr>
<tr><td>TREU 667 PC</td><td>AAATATACAAATGTAAAGAAGCTATCAGAGGTAATATAAGTGATATAAT$_N$</td><td>1152</td></tr>
</table>

<table>
<tr><th>E. ND7 Form E</th><th>Predicted mRNA Sequence</th><th>Reading Frame</th></tr>
<tr><td colspan="2"><pre>
M T T W ST
          M I M T F F M L F L V V F L H L Y R F T F G P Q
AUGACUACAUGAUAAuG*ACAuuuuuuAuGuuAuuuuuGGuAGuuuuuuuACAuuuGuAuCGuuuuACAuuuG*GUCCACAG
               ||: |#||:::|||||::|:|:|:::||||:|:||||||||||:|
              $_N$UAU-UAUAGGGAAUACGGUGAGAGUUAUCAGAGAAAUGUAAAUAAUAUA
</pre></td><td>ORF</td></tr>
<tr><td>Cell Line</td><td>gRNA Sequence</td><td>Reads</td></tr>
<tr><td>EATRO 164 PC</td><td>ATATAATAAATGTAAAGAGACTATTGAGAGTGGCATAAGGATATTAT$_N$</td><td>765</td></tr>
</table>

<table>
<tr><th colspan="2">F. ND7 Form F   Predicted mRNA Sequence</th><th>Reading Frame</th></tr>
<tr><td colspan="2">M T T W ST
M I S T F M L F L V V F L H L Y R F T F G P Q
AUGACUACAUGAUAAGUACAuuuAuGuuAuuuuuGGuAGuuuuuuuACAuuuGuAuCGuuuuACAuuuG*GUCCACAG
|||||::|:|:||:|:::||::|:||||||||||||||||
$_N$UAAAUGUAGUGAAGAUUGUCGGAGAAAUGUAAACAUAGCAUAUACA</td><td>ORF</td></tr>
<tr><td>Cell Line</td><td>gRNA Sequence</td><td>Reads</td></tr>
<tr><td>TREU 667 PC</td><td>ACATATACGATACAAATGTAAAGAGGCTGTTAGAAGTGATGTAAAT$_N$</td><td>6623</td></tr>
</table>

<table>
<tr><th colspan="2">G. ND7 Form G   Predicted mRNA Sequence</th><th>Reading Frame</th></tr>
<tr><td colspan="2">M T T W Y S I I Y V I F G S F F T F V S F Y I W S T A
AUGACUACAUGAUAuAGUAuCAuuuAuGuuAuuuuuGGuAGuuuuuuuACAuuuGuAuCGuuuuACAuuuG*GUCCACAG
|||:|||||:|||||::|:|:|:::||||:|:||||||||||:|
$_N$UAUUAUAGUGAAUACGGUGAGAGUUAUCAGAGAAAUGUAAAUAAUAUA</td><td>ARF +2</td></tr>
<tr><td>Cell Line</td><td>gRNA Sequence</td><td>Reads</td></tr>
<tr><td>EATRO 164 PC</td><td>ATATAATAAATGTAAAGAGACTATTGAGAGTGGCATAAGTGATATTAT$_N$</td><td>100761</td></tr>
<tr><td>EATRO 164 BS</td><td>ATATAATAAATGTAAAGAGACTATTGAGAGTGGCATAAGTGATATTAT$_N$</td><td>354</td></tr>
</table>

<table>
<tr><th colspan="2">H. ND7 Form H   Predicted mRNA Sequence</th><th>Reading Frame</th></tr>
<tr><td colspan="2">M T T W ST
M I S T F Y V I F G S F F T F V S F Y I W S T A
AUGACUACAUGAUAAGUACAuuuuAuGuuAuuuuuGGuAGuuuuuuuACAuuuGuAuCGuuuuACAuuuG*GUCCACAG
|||:||:||||:|||:||:||||:|:|||||||||||||
$_N$UAAGAUGCAAUGAAAGCCGUCAAGAGAAUGUAAACAUAUAAA</td><td>ARF +2</td></tr>
<tr><td>Cell Line</td><td>gRNA Sequence</td><td>Reads</td></tr>
<tr><td>EATRO 164 BS</td><td>AAATATACAAATGTAAGAGAACTGCCGAAAGTAACGTAGAAT$_N$</td><td>402</td></tr>
</table>

<table>
<tr><th colspan="2">I. ND7 Form I   Predicted mRNA Sequence</th><th>Reading Frame</th></tr>
<tr><td colspan="2">M T T W ST
M I S T M L F L V V F T F V S F Y I W S T A
AUGACUACAUGAUAAGUACAAuGuuAuuuuuGGuAGuuuuuACAuuuGuAuCGuuuuACAuuuG*GUCCACAG
||:|:|||:|::|||::||:||||:|::||||:||||||||||
$_N$UAUAGUAAGAGUCAUUGAAGAUGUGAGUAUAGUAAAAUGUAAAAUAUA</td><td>ARF +2</td></tr>
<tr><td>Cell Line</td><td>gRNA Sequence</td><td>Reads</td></tr>
<tr><td>TREU 667 PC</td><td>ATATAAAATGTAAAATGATATGAGTGTAGAAGTTACTGAGAATGATAT$_N$</td><td>12929</td></tr>
</table>

<table>
<tr><th colspan="2">J. ND7 Form J   Predicted mRNA Sequence</th><th>Reading Frame</th></tr>
<tr><td colspan="2">M T T W ST
M I S T F I V I F G S F F T F V S F Y I W S T A
AUGACUACAUGAUAAGUACAuuuAuuGuuAuuuuuGGuAGuuuuuuuACAuuuGuAuCGuuuuACAuuuG*GUCCACAG
|||||||::|:|:||:|:::||::|:||||||||||||||||
$_N$UAAAUAGUAGUGAAGAUUGUCGGAGAAAUGUAAACAUAGCAUAUACA</td><td>ARF +2</td></tr>
<tr><td>Cell Line</td><td>gRNA Sequence</td><td>Reads</td></tr>
<tr><td>TREU 927 PC</td><td>ACATATACGATACAAATGTAAAGAGGCTGTTAGAAGTGATGATAAAT$_N$</td><td>251</td></tr>
</table>

Figure 2 (extended data): Predicted alternative editing of the 5’ end of ND7.
