# Supplementary material for: Mitochondrial dual-coding genes in Trypanosoma brucei
Source: PLoS Negl Trop Dis. 2017 Oct 9;11(10):e0005989. doi: 10.1371/journal.pntd.0005989 (PMC5650466; doi:10.1371/journal.pntd.0005989)
Supplement: S3 Table — A—E: RPS12 gRNA major classes and predicted editing patterns. RPS12 terminal (5’ most) gRNA populations and the predicted mRNA sequence generated. RPS12 differs from both CR3 and ND7 in that the alternative edit that shifts the reading frame occurs just downstream of the previously identified start codon (double-underlined). We do note that the identified alternative gRNAs are rare in all of the gRNA libraries except TREU 667. (PDF) [file pntd.0005989.s005.pdf]

S3 Table: RPS12 5’ most gRNA populations and predicted mRNA sequence generated.

<table>
<tr><th>A. RPS12 Form A</th><th>Predicted mRNA Sequence</th><th>Reading Frame</th></tr>
<tr><td colspan="2">M W F L Y G C C L R F V L F V
CAAACUAAAGUAAuAuAuuAGuuuuuuGCGuAuGuGA*UUUUGUAUG*GuuGuuGuuuAC*GuuuuGuuuuAuuuGu
||||||:|:|:||:|:||||::||| |:||::|||| ||||||||
$_N$UAUAUAGUUAGAAGAUGCAUGUACU-AGAAGUAUAC-CAACAACAUAUA</td><td>ORF</td></tr>
<tr><td>Cell Line</td><td>gRNA Sequence</td><td>Reads</td></tr>
<tr><td>EATRO 164 PC</td><td>ATATACAACAACCATATGAAGATCATGTACGTAGAAGATTGATATAT$_N$</td><td>12531</td></tr>
<tr><td>EATRO 164 BS</td><td>ATATACAACAACCATATGAAGATCATGTACGTAGAAGATTGATATAT$_N$</td><td>1663</td></tr>
<tr><td>TREU 927 PC</td><td>ATATACAACAACCATATGAAGATCATGTACGTAGAAGATTGATATAT$_N$</td><td>505</td></tr>
<tr><td>TREU 667 PC</td><td>ATATACAACAACCATATGAAGATCGTGTACGTAGAAGATTGATATAT$_N$</td><td>936</td></tr>
</table>

<table>
<tr><th>B. RPS12 Form B</th><th>Predicted mRNA Sequence</th><th>Reading Frame</th></tr>
<tr><td colspan="2">M W F L Y G C C L R F V L F V
CAAACUAAAGUAAuAAAuuuuGuuuuuuuuGCGuAuGuGA*UUUUGUAUG*GuuGuuGuuuAC*GuuuuGuuuuAuuuGu
||||||:|::::||:|:|||:||||:| :||:||||:| ||||||
$_N$UAUUUAGAGUGGAAGAGACGUAUACAUU-GAAGACAUGC-CAACAAAUA</td><td>ORF</td></tr>
<tr><td>Cell Line</td><td>gRNA Sequence</td><td>Reads</td></tr>
<tr><td>EATRO 164 PC</td><td>ATAAACAACCGTACAGAAGTTACATATGCAGAGAAGGTGAGATTTAT$_N$</td><td>2218</td></tr>
<tr><td>TREU 927 PC</td><td>ATAAACAACCATACAGAAGTTACATATGCAGAGAAGGTGAGATTTAT$_N$</td><td>300</td></tr>
<tr><td>TREU 667 PC</td><td>ATAAACAACCGTACAGAAGTTACATATGCAGAGAAGGTGAGATTTAT$_N$</td><td>3834</td></tr>
</table>

<table>
<tr><th>C. RPS12 Form C</th><th>Predicted mRNA Sequence</th><th>Reading Frame</th></tr>
<tr><td colspan="2">M W F C M V V V Y V L F Y L F
CAAACUAAAGUAAAAAGuuuuuuuuuuuuGCGuAuGuGA**UUUUGUAUG*GuuGuuGuuuAC*GuuuuGuuuuAuuuGu
|||:|:|:|:|:|||::|||||:||| ||:|||||| |||:|
$_N$UUUUAGAGAGAGAAAGUGCAUAUACU--AAGACAUAC-CAAUAUAUA</td><td>ARF +1</td></tr>
<tr><td>Cell Line</td><td>gRNA Sequence</td><td>Reads</td></tr>
<tr><td>EATRO 164 BS</td><td>ATATATAACCATACAGAATCATATACGTGAAAGAGAGAGAT$_N$</td><td>144</td></tr>
</table>

<table>
<tr><th>D. RPS12 Form D</th><th>Predicted mRNA Sequence</th><th>Reading Frame</th></tr>
<tr><td colspan="2">M L F F F R M W F C M V V V Y V L F Y L F
CAAACUAAAGUAuuAuAuAAuGuuGuuuuuuuuuuCGuAuGuGA**UUUUGUAUG*GuuGuuGuuuAC*GuuuuGuuuuAuuuGu
|:|||||||||:|::|||:|:|:||:|||::|| ||||||||| ||||||
$_N$UGAUAUAUUAUAGUAAAGAGAGAGUAUAUGCU--AAAACAUAC-CAACAAGAUAUA</td><td>ARF +1</td></tr>
<tr><td>Cell Line</td><td>gRNA Sequence</td><td>Reads</td></tr>
<tr><td>TREU 927 PC</td><td>ATATAGAACAACCATACAGAAATCGTATATGCGAGAGAAATGATATTATAT$_N$</td><td>22</td></tr>
</table>

<table>
<tr><th>E. RPS12 Form E</th><th>Predicted mRNA Sequence</th><th>Reading Frame</th></tr>
<tr><td colspan="2">M W F C M V V V Y V L F Y L F
CAAACUAAAGUAAuuuAAAuuuuGuuuuuuuuGCGuAuGuGA**UUUUGUAUG*GuuGuuGuuuAC*GuuuuGuuuuAuuuGu
||||||||:|::|:|:|||::||||||||| ||:||||||| |||:|
$_N$UAAAUUUAGAGUAGAGAAAGUGCAUAUACU--AAGACAUAC-CAAUAUAUA</td><td>ARF +1</td></tr>
<tr><td>Cell Line</td><td>gRNA Sequence</td><td>Reads</td></tr>
<tr><td>TREU 667 PC</td><td>ATATATAACCATACAGAATCATATACGTGAAAGAGATGAGATTTAAAT$_N$</td><td>2664</td></tr>
</table>
